# Chronic histiocytic intervillositis (CHI): current treatments and perinatal outcomes, a systematic review and a meta-analysis

**DOI:** 10.3389/fendo.2022.945543

**Published:** 2022-07-22

**Authors:** Laurel Moar, Chloe Simela, Surabhi Nanda, Andreas Marnerides, Mudher Al-Adnani, Catherine Nelson-Piercy, Kypros H. Nicolaides, Panicos Shangaris

**Affiliations:** ^1^ School of Life Course and Population Sciences, King’s College London, London, United Kingdom; ^2^ Department of Women and Children, Guy’s and St. Thomas’ NHS Foundation Trust, London, United Kingdom; ^3^ Department of Histopathology, St. Thomas Hospital, Westminster Bridge Road, London, United Kingdom; ^4^ Harris Birthright Research Centre for Fetal Medicine, King’s College London, London, United Kingdom; ^5^ Peter Gorer Department of Immunobiology, School of Immunology and Microbial Sciences, Faculty of Life Sciences and Medicine, King’s College London, London, United Kingdom

**Keywords:** CHI, recurrent miscarriage, intervillositis, stillbirth, small gestation age (SGA)

## Abstract

**Background:**

Chronic histiocytic intervillositis (CHI) is a rare placental lesion with a high recurrence rate and poor perinatal outcomes. There are currently limited guidelines regarding the diagnosis of this condition in the index pregnancy and treatment where recurrence is suspected.

**Objective:**

The primary objective of this systematic review and meta-analysis was to determine the perinatal outcomes of pregnancies affected by chronic histiocytic intervillositis and to what extent they can be improved with treatment. The secondary objective was to assess the relationship between CHI lesion severity and pregnancy loss.

**Methods:**

A systematic search of Ovid Embase, Web of Science, Science Direct, PubMed, Ovid Medline, Google Scholar and CINAHL was carried out. Case reports, cohort, case-control and randomised controlled trials (RCT) detailing the perinatal outcomes of CHI pregnancies, both treated and untreated, were included.

**Results:**

No RCTs were identified. However, in a review population of 659 pregnancies, with additional 7 in case reports, CHI treatments included aspirin, prednisone, prednisolone, low molecular weight heparin (LMWH), hydroxychloroquine and adalimumab. A descriptive synthesis of data found mixed results for treatments in relation to live birth, miscarriage and fetal growth restriction outcomes. Furthermore, quantitative synthesis of 38 pregnancies revealed a non-significant improvement in live birth rate with CHI targeted treatment (OR 1.79 [95% CI 0.33-9.61] (p=0.50), while meta-analysis of CHI severity in line with pregnancy loss, in a sample of 231 pregnancies, revealed lower odds of pregnancy loss with less severe lesions (OR: 0.17 [0.03-0.80], p=0.03).

**Conclusions:**

This systematic review and meta-analysis reinforce notions surrounding the insufficient evidence for CHI treatment. It also strengthens previous hypotheses detailing the positive association between CHI lesion severity and odds of pregnancy loss. Aspirin, LMWH, prednisolone, hydroxychloroquine and adalimumab are candidates with varying levels of weak to moderate evidence supporting their use. Further prospective research is required to obtain robust evidence pertaining to treatment safety and efficacy and optimal drug regimes.

**Systematic Review Registration:**

[website], identifier CRD42021237604

## Introduction

Chronic histiocytic intervillositis (CHI) (also referred as CHIV) is a rare but severe placental condition characterised by the presence of an inflammatory mononuclear infiltrate in the intervillous space. CHI has been estimated to affect 6 in every 10 000 pregnancies that reach 12 weeks gestation (1, 2) and the term was first introduced by Labarrere and Mullen in 1987 as massive chronic intervillositis (3). This pathology has since been referred to by several names across the literature, including chronic intervillositis of unknown aetiology (CIUE), massive chronic intervillositis, massive perivillous histiocytosis and shortened forms - chronic intervillositis or intervillitis. Diagnostic understanding has also grown in differentiating CHI from other placental lesions through its non-infectious origin and minimal involvement of the villi or basal plate (1). Although the lesion is well described in the placental pathology literature, there is no agreed standardised diagnostic criteria or grading system for the severity of this lesion (4–7). However, the paucity of knowledge surrounding CHI biomarkers only permits retrospective diagnosis following examination of the placenta postpartum.

Furthermore, while the aetiology of CHI remains undetermined, the pathology’s high recurrence rate, reported to lie between 70 and 100% (8), highlights the need for effective treatment in future pregnancies. Some evidence of pathogenic immune mechanisms has been proposed, including an absence of the usual Th1 to Th2 immune response shift observed in normal pregnancy (1, 9–13). This raises the question of possible therapeutic benefits for immunologically based CHI treatments, with steroids and other immunosuppressants having been used in some cases.

## Objectives

This systematic review summarises the available research on treatment approaches for gravid women diagnosed with CHI in a previous pregnancy, including antithrombotic and immunosuppressive drug combinations. Primary objectives include quantifying the effectiveness of these therapies in improving perinatal outcomes compared to baseline data without treatment. Moreover, further analysis was undertaken to determine how CHI severity impacts perinatal outcomes.

## Methods

### Search strategy

PROSPERO registration for the review was fulfilled following the completion of the protocol form (CRD42021237604).

The literature search was carried out by the two reviewers, CS and LM, across seven databases: PubMed, OVID MEDLINE, Google Scholar, OVID EMBASE, Web of Science, Science Direct and CENTRAL (Cochrane Central Register of Controlled Trials) between 15^th^ February and 16^th^ February 2021. Each reviewer used the search strategy (1) comprising all known terms for CHI and a period spanning from Jan 1990 to December 2020, in three databases. CENTRAL was searched by both reviewers. Formatting adjustments were made to the search terms to optimise the strategy for each database, and email alerts were put in place to notify the reviewers of new results after the search period (**Table 1**). The two reviewers screened the search results independently, and discussions were held at both screening stages to resolve any disagreements before the final papers were decided.

**Table 1:** Search strategy for database.

No.	Science Direct, Ovid Embase, Web of Science search strategy
**1**	(((chronic histiocytic intervill*) OR (chronic intervill*)) OR (chronic intervill* of unknown?etiology)) OR (massive chronic intervill*)) OR (massive perivillous histiocyt*)
**2**	“chronic histiocytic intervillositis” [All Fields] OR “chronic histiocytic intervillositis chi” [All Fields] OR “chronic histiocytosis” [All Fields] OR “chronic histiocytosis x” [All Fields]
**3**	Search 1 OR 2

### Study selection

Selection criteria were based on the following predetermined characteristics from the protocol, starting with the population in question being identified as pregnant women of any age diagnosed with CHI in a previous pregnancy. CHI was defined as an idiopathic inflammatory lesion in the intervillous space in line with the criteria devised by Bos and colleagues (4). All other similar but distinct placental lesions were excluded, including villitis, even if in co-occurrence with CHI. Interventions described included varying doses of drug-based mono or combination therapy for CHI. However, studies without interventions were also included for comparison. Outcomes assessing the efficacy of treatment and impact of disease severity included, but were not limited to, quantitative measures such as live birth rate and birth weight. Only randomised controlled trials, cohort, case-control studies and case reports were included, with abstracts excluded. While there were no geographical limits, time constraints enforced excluding texts not in English.

### Data extraction

A modified Cochrane Public Health Group (CPHG) data extraction form was used to collect population, intervention and outcome data from the chosen papers independently by both reviewers (C.S and L.M). Additionally, each structure underwent a data checking process against the original article to detect and minimise human error. The specific information collected for each study included authors, publication year; study design, location; the number of women, number of CHI pregnancies, and number undergoing treatment. Authors were contacted to confirm information regarding population data where clarification was needed (14, 15).

### Outcome measures

Outcome measures reported in the systematic review included growth restriction and preterm birth, while those of interest in the meta-analysis were live birth rate and pregnancy loss. The preterm birth rate was defined as a live birth before 37 + 0 weeks gestation. Fetal growth restriction (FGR) or intrauterine growth restriction (IUGR) was defined as a birth weight below the 10^th^ percentile (16).

Several terms were used when determining rates of pregnancy loss in CHI. In this paper, pregnancy loss data were divided into early miscarriage (before 14 + 0 weeks gestation), late miscarriages (14 + 1 to 24 + 0 weeks gestation) and stillbirths (intrauterine death after 24 + 0 weeks gestation). Some studies also included neonatal deaths as an outcome, where this term described the loss of an infant within seven days of birth (17).

In some cases (such as with FGR), there was variation in how outcomes were classified (e.g., birth weight below 10^th^ percentile versus 3^rd^ percentile, stillbirth including pregnancy loss as early as 20 weeks versus after 24 weeks) in different studies. Such instances of outcome measure variation are indicated in the review.

### The measure of CHI lesion severity

The extent of intervillous space involvement defined CHI lesion severity as a quantifiable and standard measure. In this case, low to moderate grading referred to infiltrate occupying less than 50% of the intervillous space, and severe grading infiltrate more than 50% of the intervillous space [Parant (18), Simula (6), Sauvestre (15) but not Marchaudon (19)].

### Risk of bias and quality assessment

The risk of bias and quality of included studies was assessed independently by both reviewers using adapted versions of the Newcastle-Ottawa scale (20) and Critical Appraisal Skills Programme (CASP) checklist (21).

The 7-point system of the Newcastle Ottawa scale contained domains of selection, comparability, and outcome, while the CASP checklist aimed to detect instances of selection, measurement, classification or reporting bias. Additional consideration was given to the lack of accountability for confounding factors.

### Analysis and data synthesis

The Cohen kappa coefficient was used to assess agreement between the two reviewers at the full-text eligibility stage before generating a narrative synthesis of the findings. A high ratio indicated a reasonable level of agreement between the reviewers.

Inclusion in the meta-analysis component depended on cohort studies with a suitable differentiation of outcomes according to the presence of treatment or severity of lesions. The chosen effect measures for the meta-analyses were relative risk (RR) of pregnancy loss in moderate vs severe CHI and odds ratios (OR) for live births in treated vs untreated pregnancies, each with a 95% confidence interval and calculated based on the number of pregnancies and events. All analyses herein were carried out using RevMan (Review Manager (RevMan) Version 5.4.1, The Cochrane Collaboration 2020). Heterogeneity was measured using the I^2^ statistic, with an I^2^ >50% indicating significant heterogeneity, not due to chance. As recommended, random-effects models were used to determine the summary effect estimate where considerable heterogeneity was detected (I^2^>30%) (22).

Due to the small meta-analysis population, no additional sensitivity, subgroup, or meta-regression analyses were undertaken.

## Results

### Study selection

**Figure 1:**
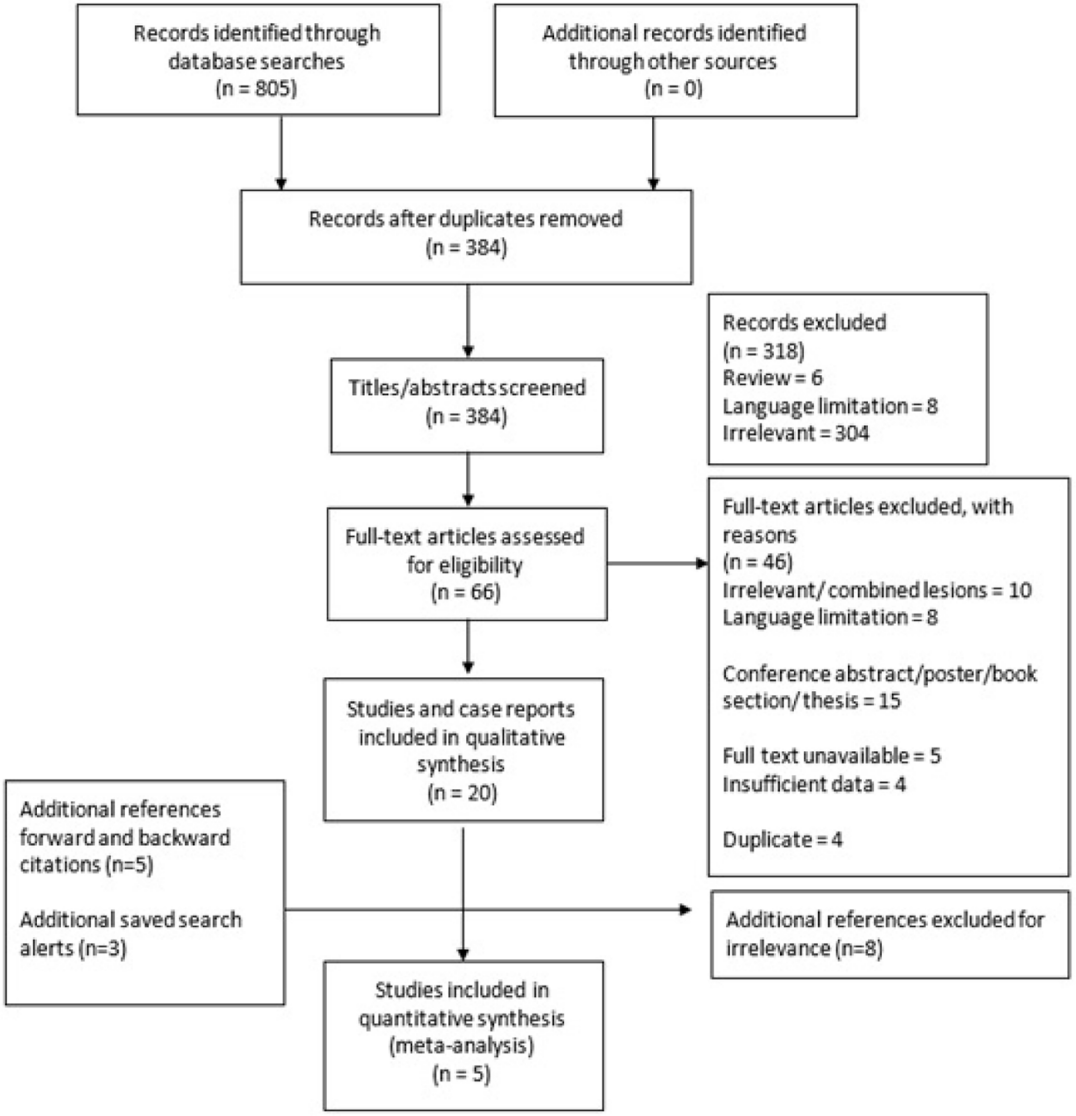
Study selection process of the systematic review and meta-analysis into outcomes of pregnancies affected by CHI.

**Figure 1** summarises (23) the study selection process and its outcomes. In total, 805 papers were found, and of these, 384 remained for the title and abstract screening once duplicates were removed. Titles and abstracts were excluded for review format, language limitation and lack of relevance leading to 66 full texts, which were narrowed down to the final 20 for systematic review inclusion. Reasons for exclusion included irrelevance, language limitation, conference abstract, poster or thesis format, full-text unavailability, and data insufficiency.

The Cohen kappa coefficient for full-text screening between the two reviewers was 0.81, indicating almost perfect agreement at this stage of study selection.

#### Study characteristics

The final twenty papers comprised 12 cohort studies and 8 case reports as no RCTs were identified. The characteristics of these mainly retrospective studies and case reports are summarised in **Table 2**. Five of the twelve cohort studies were selected for inclusion in either meta-analysis. The limited number of studies selected for meta-analysis was due to treated vs untreated outcomes being unavailable for pooling in the other seven studies.

**Table 2:** Characteristics of studies included.

Study	Location	Study design	Duration	N women	N CHI pregnancies	Mean age	Ethnicity	BMI	n/N pregnancies treated
**Doss 1995 (** 9)	USA	Case report	1974-1994	1	4	36	NR	NR	1/4
**Parant 2009 (** 18)*σ	France	retrospective cohort	2000-2006	10	14	30 (24-39)	99% white	21 (19-27)	6/14
**Marchaudon 2011** (19) ***σ***	France	retrospective	1997-2006	50	69	31 (16-43)	NR	NR	NONE
**Reus 2013 (** 11)	Netherlands	retrospective cohort study	2000-2010	22	30	31.8 (22-45)	NR	NR	NONE
**Ramya 2014 (** 24)	India	Case report	2014	1	1	22	NR	NR	NR
**Mekinian 2015 (** 25) ***σ***	France	multi-centre prospective	2011-2013	24	24	34	46% white	26	NONE
**Crawford 2016 (** 26)	Australia	Case report	2016	1	4	28	NR	NR	NONE
**Nowak 2016 (** 27)	France	retrospective cohort	1998-2010	NR	24	NR	NR	NR	NONE
**Sabra 2016 (** 28)	Spain	retrospective	2012-2016	6	6	34.83 (32-39)	83.3% European 16.7% Latinx	NR	NONE
**Ozawa 2017 (** 29)	Japan	Case report	2010-2017	1	3	29	NR	NR	1/1
**Vardi 2017 (** 30)	New Zealand	Case report	2003-2013	1	6	31	NR	NR	1
**Koby 2018 (** 31)	Canada	retrospective observational	2001-2014	29	33	Median 31 (IQR 28.5-34.5)	45.5% white, 42.4% African, 12.1% Asian	median 29.8 (26-31.7)	NONE
**Mekinian 2019 (** 32)	France	Case report	2019	2	3	38.5 (33–36)	NR	NR	2/2
**Bos 2020 (** 37)	Netherlands	Observational cohort	2000-2015	38	38	34 (24-43)	NR	NR	NONE
**Homatter 2020 (** 38)	France	retrospective case-control	2000-2016	111	111	30.8	72.1% white	NR	21/111
**Mattuizzi 2020 (** 14) **†**	France	retrospective	1997-2018	102	122	Median 32 (IQR 28-36)	61.6% white, 2% Asian 19.2% North African, 15.2% South African, 2% Hispanic	median 22	21/24
**Nohr 2020 (** 33)	Canada	Case report	2020	1	2	34	NR	NR	1/1
**Sauvestre 2020 (** 15) ***σ*†**	France	multi-centre retrospective	1997-2018	102	122	31.7	NR	23.1	NONE
**Simula 2020 (** 39) ***σ***	Canada	retrospective cohort	2006-2019	47	56	33.2	NR	25.9	10/56
**Traeder 2010 (** 40)	Germany	case series	1994-2008	4	4	34 (30-40)	NR	NR	NONE

N CHI pregnancies – only includes pregnancies diagnosed with CHI, NR – not reported, * - pregnancies with villitis excluded, all ages are means with range included in brackets if reported, ** - median and IQR used where mean and range not available, σ included in either one or both of the meta-analyses, † studies share same cohort.

Excluding case reports and series, the review population included the outcomes of 527 pregnancies affected by CHI in 439 women with a mean age of approximately 31.9 years. Fifty-eight of these cohort study pregnancies were treated, and sample size ranged from 6 to 122 pregnancies. A further 6 instances of treatment were reported in case reports and series. In total, 64 pregnancies were treated out of 554 across cohort studies, case reports and case series.

Email correspondence with authors confirmed a shared cohort between two papers, and this is highlighted in all subsequent results tables to avoid duplication and reporting errors. For review population totals, these cohorts have been counted as one (14, 15).

### Risk of bias of included studies

The risk of bias scores for each case-control and cohort study can be seen in **Table 3**. Following a judgement by both reviewers, the risk of bias was considered generally low, with an average bias score of 1.83 out of 4 across the twelve studies.

**Table 3:** Risk of bias.

Study	selection bias	measurement/classification bias	reporting bias	confounding not accounted for (no regression model)	score*
**Parant 2009 (** 18)	✓	× No mention of blinding	✓	×	2
**Reus 2013 (** 11)	✓	✓	✓	×	1
**Mekinian 2015 (** 25)	✓		✓	✓	1
**Nowak 2016 (** 27)	✓	× No mention of blinding	✓	×	2
**Sabra 2016 (** 28)	✓	× No blinding mentioned	✓	×	2
**Koby 2018 (** 31)	✓	× Investigator was aware of diagnosis no mention of blinding	✓	×	2
**Marchaudon 2011 (** 19)	✓	× Histology in conjunction with fetal autopsy so outcome known	✓	×	2
**Bos 2020 (** 37)	✓	× No mention of blinding when pathologist reviewed slides	✓	✓	1
**Homatter 2020 (** 38)	✓	× Only CHI positive cases assessed histologically	✓	×	2
**Mattuizzi 2020 (** 14)	✓	× No blinding to CHI diagnosis	✓	×	2
**Sauvestre 2020 (** 15)	× Only database search term was ‘intervillositis’	✓ All 3 pathologists blinded to diagnosis	✓	×	2
**Simula 2020 (** 39)	× Database search was for CIUE and chronic intervillositis	×	✓	×	3

*Risk of bias score is out of a total of 4.

✓ Low risk of bias.

× High risk of bias.

Selection bias was reported in four of the twelve studies due to how participants were selected from a pathology database (15, 18, 37, 39). Furthermore, in other cases, selection bias was detected because of limited search terms comprising only of ‘CIUE and chronic intervillositis’ in one study and ‘intervillositis’ in another (15, 39).

Seven studies indicated measurement/classification bias in diagnosis and grading of CHI, whereby pathologists were aware of and not blinded to the previous CHI diagnosis.

Reporting bias was undetected. However, ten out of twelve studies did not state accountability for confounding variables.

Study quality assessment outcomes are shown in **Table 4**. High scores were obtained in the selection domain, with participants seen as broadly representative of the population in question. The area in which numerous studies were deficient was comparability and outcome measures. Lack of differentiation between early and late miscarriage outcomes and methods that did not describe the assessment of lesion severity as a possible mediator of perinatal outcome was also suboptimal. Furthermore, lack of information on population comorbidities or medication also led to lower quality scores in the outcome domain.

**Table 4:** Quality assessment using Newcastle-Ottawa scoring system.

Study	year	Study design	Selection (3)	Comparability (2)	Outcome (2)	total (7)	Comment
**Parant 2009 (** 18)	2009	retrospective cohort	3	2	1	6	No details of whether pathologists were blinded
**Marchaudon 2011 (** 19)	2011	retrospective	3	1	1	5	Pathologists carried out histological analysis in conjunction with autopsy - no blinding, no mention of medications
**Reus 2013 (** 11)	2013	retrospective	3	1	2	6	
**Mekinian 2015 (** 25)	2015	prospective	3	2	2	7	
**Nowak 2016 (** 27)	2016	retrospective cohort	3	2	1	6	
**Sabra 2016 (** 28)	2016	retrospective	2	1	1	4	No blinding mentioned, and only 6 patients
**Koby 2018 (** 31)	2018	retrospective observational	3	1	1	5	Pathologist was aware of diagnosis, no details on medication
**Bos 2020 (** 37)	2020	observational cohort	3	1	1	5	No comment on whether pathologists were blinded to prior diagnosis.
**Homatter 2020 (** 38)	2020	retrospective case-control	2	1	2	5	Early miscarriage excluded
**Mattuizzi 2020 (** 14)	2020	retrospective	3	1	1	5	No comments on anyon any blinding
**Sauvestre 2020 (** 15)	2020	retrospective	2	1	1	4	Only histological database search term was ‘intervillositis’, live birth outcomes unclear in table, 10 placentas missing
**Simula 2020 (** 39)	2020	retrospective cohort	3	2	1	6	No comment on whether pathologist was blinded

### Treatment combinations

None of the cohorts included in the systematic review was fully treated. The proportion of the four cohorts receiving targeted treatment for CHI ranged from 18-88%, and the other eight cohorts were labelled as untreated. **Table 5** summarises the treatment combination details in the treated pregnancies.

**Table 5:** Treatment combinations used in case pregnancies affected by CHI.

**Author/Year**	**Intervention details**		
	**N**	**Untreated**	**Treatment details**	
**Parant 2009 (** 18)	14	8	aspirin (n=4) aspirin + corticoid (n=2)	
**Traeder 2010 (** 40)	4	4	NONE	
**Marchaudon 2011 (** 19)	69	69	NONE	
**A. Reus 2013 (** 11)	30	30	NONE	
**Mekinian 2015 (** 25)	24	3	aspirin/LMWH (n=4) aspirin + prednisone (n=6) aspirin+ prednisone+ LMWH (n=5) aspirin +prednisone+ LMWH+ hydroxychloroquine (n=6)	
**Nowak 2016 (** 27)	24	24	NONE	
**Sabra 2016 (** 28)	6	6	NONE	
**Koby 2018 (** 31)	33	33	NONE	
**Bos 2020 (** 37)	38	38	NONE	
Homatter 2020* ( 38)	111	90	aspirin (n=18) LMWH (n=7) corticosteroids(n=6)	
**Mattuizzi 2020 (** 14)	122	122	NONE	
**N.K Simula 2020 (** 39)	56	37	aspirin 81mg +dalteparin 7500 IU+ hydroxychloroquine 400mg (n=2) aspirin 81mg (n=3) dalteparin (n=1) prednisone (n=1) aspirin + dalteparin (n=3)	
**Sauvestre 2020 (** 15)	122	122	NONE	

* in combination but assessed separately.

There was a variation of gestational ranges when the treatment was commenced. Those containing aspirin were the most common of the treatment regimes, followed by low molecular weight heparin (LMWH) and finally corticosteroid regimes (prednisone/corticoid), either alone or in combination.

### Outcomes

**Table 6** summarises the perinatal outcomes of these studies. Continuous variables are reported as means followed by the standard deviation and range in brackets if available.

**Table 6:** Perinatal outcomes for CHI pregnancy cohorts.

Study	Year	n/N pregnancies treated	Live Birth Rate	Avg. Live Birth weight (g)	Gestational age at delivery (weeks)	Spontaneous preterm live birth	FGR(<10^th^ centile) live or not	Miscarriage < 14 weeks	Miscarriage 14 -24 weeks	Still birth IUD > 24 weeks
**Parant 2009 (** 18)	2009	6/14	5/14	1550 (590-2760)	34.6 (27.5-37)	10/10	8/11	2/14	1/14	2/14
**Traeder 2010 (** 40)	2010	NONE	4/4	995 (495-1640)	31.2 (27-35.6)	4/4	3/4 (<10th percentile) 2/4 (<3rd percentile)	NO	NO	NO
**Marchaudon 2011 (** 19)	2011	NONE	21/69	1780 ± 590 (920-2830)	35.0 ± 2.5 (30.6-39.0)	13/21	24/69 (<3rd percentile)	21/69 (<12 weeks)	9/69 (12-22 weeks)	18/69 (>22 weeks)
**Reus 2013 (** 11)	2013	NONE	10/30	NR	NR	6/10	NR	4/30 (<12 weeks)	2/30 (12-16 weeks)	NR
**Mekinian 2015 (** 25)	2015	21/24	16/24	2493 ± 678	38 ± 1.6	5/16	3/24	4/24 (<10 weeks)	4/24 (>10 weeks)	NR
**Nowak 2016 (** 27)	2016	NONE	17/24	1634 (610-2875)	34.3± 3.7	11/17	14/21	/1/24	2/24	2/24
**Sabra 2016 (** 28)	2016	NONE	2/6	1695 (1290-2100)	35.5 (33-38)	1/2	2/6	4/6	NO	NO
**Koby 2018 (** 31)	2018	NONE	20/33	NR	NR	16/20	23/33 (<10th percentile), 15/33 (<3rd percentile)	2/33 (<20 weeks)	NR	11/31 (>20 weeks)
**Bos 2020 (** 37)	2020	NONE	31/38	NR	NR	21/31	16/38 (<3rd percentile)	NR	1/38	3/38
**Homatter 2020 (** 38)	2020	21/111	62/111	1500 ± 885	33.6 ± 4.7	4/62	62/111 (<3rd percentile)	NR	6/111 (14-21 weeks)	22/111 (22-42 weeks)
**Mattuizzi 2020 (** 14)	2020	NONE	70/122	NR	NR	38/70	81/122	17/122	17/122	NR
**Simula 2020 (** 39)	2020	10/56	3/56	NR	NR	1/6	7/15 at 20 weeks	29/56 (<12 weeks)	13/56 (12-22 weeks)	6/56 (>23 weeks)
**Sauvestre 2020 (** 15)	2020	NONE	70/122	NR	NR	5/70	46/70 (alive)	17/122	1/122	16/122
**Total**		58	336/659	1679.68	34.47	135/336	289/424	101	56	80

Live birth rate is as a fraction over total CHI pregnancies in each study, * pregnancies with villitis excluded, NR – not reported, average birth weight and gestational age at delivery with standard deviation if reported and range in parentheses. Parentheses in miscarriage and FGR indicate differing classification criteria.

#### Live births

The live birth rate varied considerably between studies, ranging from 30.4% to 100%. Interestingly, neither of the cohorts displaying results at these extremes were indicated as receiving target CHI treatment (19, 40). There was also variance within similar populations, as several of the papers were based within the same countries yet yielded different live birth rates for example the study by Bos et al., 2020 reported a live birth rate more than twice as high as that reported by Reus et al., 2013 (11, 37). Comparable live birth rates of 67% and 70% were observed in two studies with a similar cohort size despite 88% of one cohort receiving targeted treatment (25) versus none of the other (27). Overall, the average live birth rate for cohorts in which no treatment was stated was 58.3% versus 40.9% in cohorts with a partial treatment.

#### Birth weight

Data on average live birth weight were available in seven studies, three of which had a proportion receiving treatment. In most cases, this outcome rarely reached 2500g however, the highest mean birth weight of 2493g was achieved in one study in which 88% of the cohort received treatment (25). Furthermore, a low average birth weight of 995g was reported by Traeder and colleagues (40) across a case series of four untreated pregnancies. However, this positive treatment effect was not always reflected, with higher birth weight observed in untreated cohorts (19, 27, 28) compared to partially treated cohorts (18, 38).

#### Preterm birth

In five out of six studies, a low average birth weight was accompanied by an average gestational age at delivery <37+0 weeks. The general trend towards high preterm birth in both untreated and treated CHI pregnancies is also evident in a preterm birth rate of 40.2% across the twelve studies. Interestingly, the study by Mekinian and colleagues in which the largest proportion of the cohort was treated (88%) had a lower preterm birth rate of 31.25% (25). However, this was not the case for all partially treated cohorts, which, for the most part, had a lower mean gestational age at delivery (38) and higher preterm live birth rate than the untreated cohort (19, 27, 28).

#### Fetal growth restriction

Many infants were small for gestational age. Studies reported fetal growth restriction of below 10^th^ centile (with some even below 3^rd^ centile) in both live and stillborn or neonatal death groups.

Similar prevalence of FGR < 10^th^ centiles was reported in two studies (66.7% and 69.7%, respectively), both of which had entirely untreated cohorts (27, 31). In treated cohorts, this same variable ranged from 12.5% (25) to 72.7% (18)^19^ with treated proportions being 88% and 42.8%, respectively.

Severe FGR, <3^rd^ centile, was only reported as an outcome in 5 studies (19, 31, 37, 40). Of these, one study had an 18.9% treated cohort with a severe FGR prevalence of 55.9% (38). Four untreated study cohorts have reported an occurrence rate of severe FGR as 69.7%, 42.1%, 34.8% and 50% (19, 31, 37, 40) respectively.

#### Miscarriage, stillbirth and neonatal death

Overall, miscarriage rates ranged between 2.63% and 20% in untreated cohorts (11, 37). The miscarriage rate was much wider in treated cohorts (range between 5.4% and 75%). In some cases, miscarriage timing was before 14 weeks rather than after 14 weeks, but this was only in five out of the twelve cohorts (11, 15, 18, 28, 39).

Stillbirth rates varied between 10.7% and 19.8% in treated pregnancies (38, 39). Like miscarriage rates, this was comparatively higher than the untreated cohort range of 7.9% to 13.1% (15, 22). Interestingly, there were also instances of no reported stillbirths in untreated populations with small sample sizes (28, 40).

#### Neonatal death

Neonatal death was not widely reported as an outcome and ranged from 1.11% up to 23.3% across four studies (11, 14, 15, 38). There are no reported cases in both partially treated and untreated cohorts (18, 27).

#### Individual pregnancy outcomes

As shown in **Table 7**, individual patient outcomes were available from seven case reports, one case series, and two retrospective cohort studies that reported individual patient details. 21 of the 31 case pregnancies were treated, and 9 of these resulted in a live birth, of which 4 were at term. This is compared to 6 out of 10 untreated pregnancies resulting in live births and 1 of these 6 occurring at term.

**Table 7:** Individual patient outcomes in pregnancies affected by CHI.

Author/Year	Maternal Age	G	P	No. previous CHI pregnancies	Case Pregnancy Treatment	Treatment duration	Case Pregnancy Outcome
**Doss 1995 (** 9)	36	13	2	4/12	Prednisone	NR	Live birth, 2170g
**Parant 2009 (** 18) **a**	35	8	5	NR	None listed	–	Miscarriage 14 weeks
**Parant 2009 (** 18) **b**	25	5	0	NR	Aspirin/corticoid (prednisone 20mg)	Full gestation	Miscarriage 8 weeks
**Parant 2009 (** 18) **c**	29	2	1	NR	None listed	-	Live birth, 2310g, 34 weeks
**Parant 2009 (** 18) **d**	31	2	1	NR	None listed	-	IUFD (Intrauterine fetal death), 180g, 23 weeks severe IUGR
**Parant 2009 (** 18) **e**	31	2	1	NR	None listed	-	TOP, 190g fetal weight, 22 weeks, severe IUGR
**Parant 2009 (** 18) **f**	29	1	0	NR	None listed	-	Live birth, 2080g, 37 weeks, severe IUGR
**Parant 2009 (** 18) **g**	24	4	0	NR	Aspirin	Full gestation	Miscarriage 10 weeks
**Parant 2009 (** 18) **h**	25	2	0	NR	Aspirin	Full gestation	IUFD, 330g, 26.5 weeks FGR
**Parant 2009 (** 18) **i**	31	3	1	NR	Aspirin/corticoid (prednisone 5mg)	Full gestation	TOP, 215g, 22.5 weeks, FGR
**Parant 2009 (** 18) **j**	28	3	2	NR	Aspirin	Full gestation	Live birth, 2760g, 37 weeks
**Parant 2009 (** 18) **k**	27	2	1	NR	Aspirin	Full gestation	Live birth, 2320g, 37.5 weeks, IUGR
**Parant 2009 (** 18) **l**	33	3	2	NR	None listed	-	TOP, 392g fetal weight, 26 weeks, FGR
**Parant 2009 (** 18) **m**	25	3	0	NR	None listed	–	Live birth, 590g, 27.5 weeks, FGR
**Parant 2009 (** 18) **n**	31	2	0	NR	None listed	-	IUFD, 160g, 28.5 weeks, FGR
**Traeder 2010 (** 40) **a**	30	2	2	NR	NONE	NONE	Live birth, 1640g, 32 weeks
**Traeder 2010 (** 40) **b**	40	4	1	NR	NONE	NONE	Live birth FGR, 1440g, 35 weeks
**Traeder 2010 (** 40) **c**	30	1	1	NR	NONE	NONE	Live birth FGR, 405g, 29 weeks
**Traeder 2010 (** 40) **d**	36	1	1	NR	NONE	NONE	Live birth FGR, 495g, 27 weeks
**Ramya 2014 (** 24)	22	1	0	NR	None listed	–	IUD 460g, 24 weeks
**Crawford 2016 (** 26)	28	5	1	3/4	None listed	-	TOP 21 weeks
**Ozawa 2017 (** 29)	29	7	0	2/6 **	Prednisolone 20mg + LDA	4 - 29 weeks	Live birth, 2051g, 35 weeks
**Vardi 2017 (** 30)	31	6	0	3/5	20mg oral prednisone OD, 40mg LMWH (enoxaparin) subcutaneously OD, aspirin 100mg OD	Prednisone conception-20weeks and decreasing dose to 28 weeks; LMWH 6 weeks onwards	Live birth, 2200g, 34 weeks
**Mekinian 2019 (** 32) **a**	37	13	1	NR	Adalimumab + aspirin 100mg/day + prednisone 10mg/day	2 months preconception - 9 weeks gestation	Live birth, 2960g 38 weeks
**Mekinian 2019 (** 32) **b**	40	NR	NR	NR	Adalimumab 40mg every 2 weeks subcutaneously	2 months prior to oocyte donation - 9 weeks gestation	Live birth, - g, 39 weeks
**NK Simula 2020 (** 39) **a**	NR	6	1	5/5*	Aspirin 81mg daily, dalteparin 7500 IU daily, hydroxychloroquine	From positive pregnancy test - delivery	Live birth, -g, - weeks
**NK Simula 2020 (** 39) **b**	NR	NR	NR	NR	Aspirin 81mg daily	From positive pregnancy test - delivery	Live birth at term, - g
**NK Simula 2020 (** 39) **c**	NR	NR	NR	NR	Aspirin 81mg daily	From positive pregnancy test - delivery	Pregnancy loss
**NK Simula 2020 (** 39) **d**	NR	NR	NR	NR	Aspirin 81mg daily	From positive pregnancy test - delivery	Pregnancy loss
**NK Simula 2020 (** 39) **e**	NR	NR	NR	NR	Dalteparin 5000-7500 IU daily	From positive pregnancy test - delivery	Pregnancy loss
**NK Simula 2020 (** 39) **f**	NR	NR	NR	NR	Prednisone	From positive pregnancy test - delivery	Pregnancy loss
**NK Simula 2020 (** 39) **g**	NR	NR	NR	NR	Aspirin 81mg daily, dalteparin 5000-7500 IU daily	From positive pregnancy test - delivery	Pregnancy loss
**NK Simula 2020 (** 39) **h**	NR	NR	NR	NR	Aspirin 81mg daily, dalteparin 5000-7500 IU daily	From positive pregnancy test - delivery	Pregnancy loss
**NK Simula 2020 (** 39) **i**	NR	NR	NR	NR	Aspirin 81mg daily, dalteparin 5000-7500 IU daily	From positive pregnancy test - delivery	Pregnancy loss
**NK Simula 2020 (** 39) **j**	NR	NR	NR	NR	Aspirin 81mg daily, dalteparin 7500 IU daily, hydroxychloroquine	From positive pregnancy test - delivery	Ongoing?
**Nohr 2020 (** 33)	34	4	1	2/3	IVIg therapy, prednisone, LMWH, Aspirin	NR	IUGR, IUFD 25 weeks

Live birth rate as a fraction over total CHI pregnancies in study, * 3/5 treated with Aspirin 81mg daily, dalteparin 5000-7500 IU daily, prednisone 10-40mg daily; 1/5 treated with aspirin 81mg daily, dalteparin 7500 IU daily, hydroxychloroquine 400mg daily.** pregnancy 5 treated with aspirin 81mg/ day resulted in IUD and IUGR 210g at 17 weeks; pregnancy 6 treated with aspirin 81mg/day, heparin 10 000-15 000IU/day IUGR 33 weeks 1032g.

### Meta-analysis

**Figure 2:**
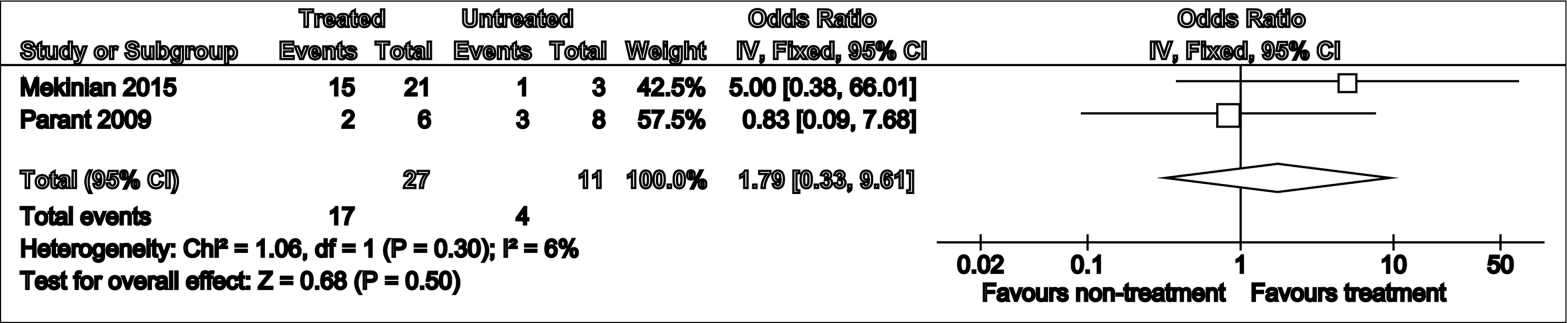
Forest plot summarising odds of live birth rates in treated vs untreated pregnancies.

Meta-analysis of live birth outcomes in two studies in which treated and untreated data was available revealed a non-significant improvement in live birth rates with treatment (Odds Ratio: 1.79 [0.33-9.61], p=0.50) (**Figure 2**). The pooled population included 27 treated and 11 untreated pregnancies. Heterogeneity (I^2^) was estimated to be 6%, using fixed effect model.

**Figure 3:**
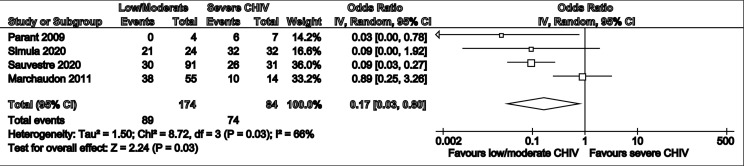
Forest plot summarising risk ratios of pregnancy loss in low/moderate vs severe CHI.

Meta-analysis of pregnancy loss in relation to CHI severity across four studies with a pooled population of 174 low to moderate severity cases and 84 severe cases indicated significantly lower odds of pregnancy loss in cases with less severe lesions vs those with increased severity (Odds Ratio: 0.17 [0.03-0.80], p=0.03) (**Figure 3**). Largely homogenous grading criteria in all four studies permitted low to moderate lesions to be defined as <50% intervillous infiltrate involvement and >50% leading to a severe classification (supplementary data file).

## Discussion

### Principle findings

The primary objective of this systematic review and meta-analysis was to identify and quantify the effectiveness of current treatment regimens for pregnant women diagnosed with CHI following a previous pregnancy. Effectiveness was quantified by comparing perinatal outcomes, including live birth, miscarriage, stillbirth rates and neonatal death in treated and untreated pregnancies, birth weight (normal, IUGR), as well as preterm birth rate, with a positive effect in such measures indicating effective treatment.

The commonly used treatment regimens for CHI, either as standalone or as combination therapy include aspirin, prednisone, low molecular weight heparin, biological agents most commonly adalimumab and hydroxychloroquine (**Table 8**). The partially treated cohorts often performed equally or even worse in the outcome domains of live birth rates, preterm birth, fetal growth restriction, miscarriage and stillbirth rates. Indeed, the pooled treatment effect for live birth rates in treated vs untreated pregnancies produced an odds ratio of 1.79 [95% CI 0.33-9.61] (p=0.50). This converts to a risk ratio of 1.4 [95% CI 0.48-4.05] (p=0.54), which reveals the likelihood of live births in the treated group was 1.4 times that in the untreated, although this was not statistically significant. Taken collectively, this suggests that antithrombotic and immunosuppressive treatment for CHI cannot be significantly effective in improving perinatal outcomes in affected pregnancies. However, classifying these results as clinically insignificant due to exceeding the arbitrary cut off p=0.05 would be misleading as such a small, pooled population of 27 treated and 11 untreated pregnancies suggests a degree of imprecision. Consequently, we can conclude that these findings are inconclusive, and there is insufficient evidence of a treatment effect, as opposed to proof of no treatment effect (34).

**Table 8:** Summary of CHI treatments, their indication in mild, moderate, or severe cases and the strength of evidence surrounding each.

Treatment	Dose	Indication	Strength of Evidence
**Aspirin**	75-100mg	Low, moderate, severe	Moderate
**Dalteparin**	———	Low, moderate, severe	Moderate
**Enoxaparin**	———	Low, moderate, severe	Moderate
**Prednisone/Prednisolone**	10-20mg	Low, moderate, severe	Moderate
**Adalimumab**	———	Moderate to severe	Weak
**Hydroxychloroquine**	———	Moderate to severe	Weak
**Tacrolimus**	———	Moderate to severe	Weak

The secondary objective of this review was to determine the extent to which CHI severity – measured as a percentage of intervillous space occupied by infiltrate- impacts perinatal outcomes. It was anticipated that establishing a relationship between progression of placental pathology, in subsequent pregnancies, and extent of adverse outcomes would improve understanding of treatment potential as seen by the effect of treatment (monotherapy or combination therapy) on the placental tissue. This in turn would help to explore if improvements in lesions (with or without complete remission) positively correlate with more favourable perinatal outcomes. Results demonstrate a small severity effect in relation to pregnancy loss (Odds Ratio: 0.17 [0.03-0.80], (p=0.03). The larger pooled population of 174 low or moderately affected pregnancies and 84 pregnancies affected with severe CHI infers a more reliable evidence that any improvement in the severity of lesions correlates to a reduction (albeit small) in the odds of miscarriage or stillbirth.

The proposed hypothesis around the effect of treatment regimens is the reduction in the severity of lesions through targeted anti-inflammatory, immunosuppressive and anti-thrombotic mechanisms, which in turn would help improve the perinatal outcome.

The discrepancies in severity classification systems between papers cannot be ignored. It is known that CHI can exist with or without fibrin deposition and so while it is not necessary for diagnosis, the role of fibrin in severity classification systems is interesting. While in some, fibrin was acknowledged, this was not the case in studies with methods that did not call for fibrin to be noted (19, 39). **Table 9** illustrates the varying inclusion and exclusion criteria for CHI classification in all the studies included.

**Table 9:** Different diagnostic criteria for CHI among included studies in which it was stated.

	Doss 1995 (9)	Parant 2009 (18)	Traeder 2010 (40)	Marchaudon 2011 (19)	Reus 2013 (11)	Ramya 2014 (24)	Mekinian 2015 (25)	Nowak 2016 (27)	Sabra 2016 (28)	Vardi 2017 (30)	Koby 2018 (31)	Bos 2020 (37)	Homatter 2020 (38)	Matuizzi 2020 (14)	Simula 2020 (39)	Nohr 2020 (33)	Sauvestre 2020 (15)
**Inclusion**																	
Infiltrate in intervillous space	+	+	+	+	+	+	+	+	+		+	+	+	+	+	+	+
Mononuclear infiltrate	+	+		+	+	+	+	+	+		+	+	+	+	+	+	+
Histiocytic CD68+ marker			+			+	+		+								+
Massive/diffuse infiltrate				+			+						+				
Fibrin deposits	+	+			+								+/-				
Trophoblastic necrosis	+																
Maternal origin of infiltrate																	
**Exclusion**																	
Signs of infection						+	+		+			+		+	+		+
Villitis																	
Presence of other placental lesions			+														
Malaria						+	+		+								

In retrospective studies, it was not possible for all placentas in the database to be examined for CHI. Therefore, many relied on the assumption that CHI placentas had accurately been documented as such and the search terms used to recover them were adequate (38).

Furthermore, there was an invariable absence of adjustment for confounders known to increase risk of adverse perinatal outcomes such as maternal BMI and smoking which at times, were not measured during the process of data gathering in some cases or noted in many of the case reports.

### Comparison with existing literature

Overall, the findings of this review were consistent with those previously available evidence. For instance, the strong association between FGR, miscarriage, and stillbirth reflected in **Table 6** (2). FGR frequency <10^th^ percentile, was estimated at 61% across nine studies in our review population compared to 48% in a previous meta-analysis (35). The total population in our review did not confirm a higher prevalence of early miscarriage compared to late, as was seen by Rota and colleagues (5), however it was found that there was a higher rate of intrauterine deaths in the remit of early miscarriage rather than stillbirth or late miscarriage. Nevertheless, the relatively high incidence of either early or late pregnancy loss emphasises the impact of placental insufficiency without maternal vascular malperfusion on increased adverse pregnancy outcomes.

Interestingly, our conclusion that increased CHI lesion severity correlates with increased risk of pregnancy loss disagrees with the findings of the most prominent prospective study in the area (25) which states that maintenance of visible CHI pathology is not always indicative of adverse outcomes. However, this study did find that the absence of these lesions was linked to more positive outcomes. Further investigation may be required to find a more definitive answer. It is worth acknowledging here that there has previously been a lack of international consensus on indications for sending placental pathology (and for reporting by perinatal pathologists), following an adverse pregnancy outcome, and therefore CHI itself may be under-reported. However, there has been a reform in this area more recently due to the creation of the international Amsterdam consensus criteria in 2016 (36).

The lack of significant treatment effects in CHI, though cautiously interpreted by our review, was also highlighted by Contro et al (41). This 2010 review on CHI concluded that treatments investigated at the time had no significant, and even a detrimental, effect on perinatal outcome. Our review provides evidence supporting this conclusion, despite numerous novel studies conducted in the 10-years since the last systematic review (41). However, one of the explanations for this may be a lack of consensus about the case selection for treatment, gestation of commencing treatment (and stopping), and treatment regimens (monotherapy or combination therapy). Further explanation for individual worse outcomes in combination therapy pregnancies has been highlighted by Mekinian et al., pertaining to confounding by indication and severity – whereby pregnancies with worse prognoses (maternal history of previous IUD) are targeted with combination treatment, while those with prognostically better outcomes are not (25).

Historically, there has been a lack of evidence around the generic use of immunosuppressive therapies in the management of recurrent pregnancy loss (42). A previous study, albeit with suboptimal case selection and design, highlighted a possible association between first-trimester prednisone exposure and cleft lips and palates in infants (43). This has since been disputed (44), and there is considerable experience in the use of oral steroids for many a condition in pregnancy, including lupus, transplants, severe asthma, etc. Nevertheless, long term use of steroids has been linked with increased preterm delivery rates (secondary to preterm prelabour rupture of membranes), neonatal intensive care admission and low birth weights in the neonates, and increased chance of maternal dependence on steroids and steroid induced diabetes (and its associated complications) (45–47). While it is not clear whether any of these adverse outcomes were documented as a result of oral steroids like prednisone in our review studies, its use in the management of pregnancies previously affected by CHI as a part of off-label treatment regimes in the UK, in order to optimise pregnancy outcome (improve live birth rate, and prolong gestation at delivery), is still surrounded by controversy (48).

Antithrombotic therapy is relatively effective in treating pregnancy loss, particularly in those with associated antiphospholipid syndrome (42, 46). A recent Cochrane review concluded that combined treatment of aspirin with heparin is more likely to lead to higher birth rates in women with recurrent pregnancy loss associated with antiphospholipid antibodies, compared to aspirin alone (49). Though still yet to be confirmed, this offers a potential means of treating the proposed antibody mediated and pro-inflammatory pathophysiology of CHI. A recent review drew upon the application of immunomodulatory drugs in autoimmune conditions during pregnancy to emphasise the safety and merit of hydroxychloroquine in CHI (50).

Additionally, CHI has been associated with maternal hypertensive disorders in some studies but evaluating this was outside the remit of this paper (8). Antithrombotic therapy such as aspirin is also cited by The American College of Obstetricians and Gynaecologists as appropriate in the prevention of fetal growth restriction, which in some cases is due to placental insufficiency (16, 51). Hence, despite further research being warranted, the practice of the use of aspirin and heparin in the management of pregnancies affected by CHI, is not discordant with current practice. Anti-thrombotic drugs such as unfractionated and low molecular weight heparin, adjunctive low dose aspirin (with or without other immunosuppressive agents such as hydroxychloroquine, azathioprine, adalimumab, tacrolimus) currently form the basis of off-label CHI treatment in the UK (48). A 2021 study by Brady and colleagues has however offered some promising results in the application of hydroxychloroquine and prednisolone (in conjunction with mainstay aspirin and heparin) in improving CHI lesion severity and bringing about a 62.3% reduction in subsequent pregnancy loss (52). The study reported lower FGR, preterm, stillbirth and neonatal death rates in treated pregnancies although statistical significance was restricted by the small sample size (52).

Co-occurrence of placental lesions was encountered as frequently as 30% in one study and 25% in another (5, 18). While these cases were beyond the scope of the review, the reality of coexisting pathology is worth recognising (50). In one study, combined lesions formed a considerable proportion of placental samples (35%). Although these were excluded from our data, the debate on viewing combined lesions as separate entities continues (27). The application of aspirin and corticosteroids in a pregnancy affected by both villitis and CHI was documented as having a positive outcome in one case report (53). It is questionable whether the same treatment effect would be observed in pregnancies affected by other placental lesions adjunct to CHI, and further research into this should be encouraged. We stress the value of a multidisciplinary discussion involving perinatal pathologists in such cases, where there are such services available, to have a case-based consensus of treatment regimes (36).

### Strengths and limitations

This is a rare condition. As discussed above, there is a lack of consensus and guidance on indications for placental pathology following pregnancy loss or adverse pregnancy outcomes. Not all placental pathology is assessed by perinatal pathologists. Therefore, it can be acknowledged that the actual incidence of CHI is underreported. There is only a small amount of literature on CHI, and it is unlikely that any relevant studies were omitted through the search strategy. It has been noted that observational study titles can be misleading and require full-text screening for relevance (22). The manageable number of search results enabled manual screening and several checking processes to be carried out to ensure that no relevant studies were missed. Saved search alerts permitted new papers released after the search period to be identified and screened equally.

However, it must be acknowledged that there was an enforcement of an English-language criterion due to time constraints, and it is possible that this introduced a level of selection bias to this systematic review and meta-analysis.

Lastly, not all relevant data, i.e., treated vs untreated outcomes, appeared to be included in studies, and although raw data were requested through email correspondence, this was not available. As a result, only 38 untreated and treated cases across two studies could be included in the meta-analysis for treatment effect concerning live birth rates specifically, and while data from individual studies appeared to favour untreated cohorts, pooled data in the meta-analysis revealed a non-significant improvement in treated outcomes may suggest publication bias. This is likely due to the exclusion of multiple studies from the meta-analysis. There was a lack of data distinguishing treated vs untreated outcomes in all these other partially treated cohorts (18, 25).

Associated maternal outcomes like hypertensive disorders in pregnancy and treatment-related adverse effects like steroid-induced diabetes have been noted in case reports and from experience with our group. However, evaluating this was outside the remit of this review (54).

## Conclusion

This review examined the outcomes of 554 pregnancies, 64 of which were treated by either aspirin, prednisolone or LMWH alone or in conjunction. Additional therapies included hydroxychloroquine and adalimumab. Based on efficacy being defined as significantly reducing the prevalence of adverse perinatal outcomes in affected pregnancies, the findings have further strengthened the available evidence that there is no known effective treatment for CHI in pregnancy. The paucity of research using comparison groups in a case-control design has led to challenging analysis and equivocal results. Gaining a deeper insight into novel, effective therapeutics will require international collaboration due to the rare nature of this pathology.

Current therapies for the general treatment of recurrent pregnancy loss form a basis for building these next steps for CHI treatment. Existing recommendations support the use of antithrombotic therapies more than immunosuppressants. The likely need for combination therapy in the event of previous IUFD has been highlighted (25), and this review showed the general value of combination therapy in current practice. Novel therapies not previously reviewed, such as adalimumab and hydroxychloroquine, warrant further research as uncertainty surrounding their efficacy and safety has led to their application only informally recommended in moderate to severe cases.

Compared to other lesions, the high recurrence rate and more severe perinatal outcomes associated with CHI underscore the value of a precise differential diagnosis. Further research may permit a stepwise approach utilising aspirin, heparin, and hydroxychloroquine to form the basis of individualised treatment plans with cost/benefit analysis in line with the previous pregnancy outcomes and multidisciplinary treatment counselling.

Fundamentally, research needs to address the aetiology of CHI to gain a complete understanding of possible ways to treat it. Once this is achieved, observational studies are of value, and even if still retrospective, augmentation of database results with additional hospital data will help account for confounding. Our review only identified one complete and one ongoing prospective multi-centre study. A prospective registry of women with pregnancies previously affected by CHI, involving non-profit organisations like CHI support (48), who may provide ongoing support to these families, may help with a holistic multidisciplinary approach to the management of these pregnancies and improve our understanding of this rare pathology. Equally, a prospective multi-centre design should be the objective model of future studies with additional outcome measures such as Apgar Scores and NICU admissions and later achievement of neurodevelopmental milestones.

Interventions should include, but not be limited to, aspirin, prednisone, and heparin, with or without other immunosuppressive agents such as hydroxychloroquine, azathioprine, adalimumab, tacrolimus). Researchers should be attentive to side effects caused by therapies and their comparative effectiveness in combination or alone. This will help develop future guidelines for CHI treatment, and hopefully, long-term follow-up data regarding childhood outcomes in untreated versus treated CHI pregnancies will also become available.

The best effort to improve pregnancy outcomes in pregnancies associated with CHI is surveillance and identification of screening tools in the index pregnancy, where CHI is suspected. Equally, where CHI has been previously identified, screening tools may help increase surveillance and tailor treatment modalities and regimens. Use of first-trimester markers (55, 56) like placental growth factor or second-trimester markers like alkaline phosphatase, ultrasound markers like increased uterine artery pulsatility index and reduction in amniotic fluid in the third trimester, and use of fetal placental MRI using diffusion imaging have all been reported either alone or in conjunction with each other. Although a single sufficiently specific biomarker is yet to be identified (31), further research into these potential prognostic biomarkers would be invaluable (54) in reducing the prevalence of adverse perinatal outcomes in this pathology by permitting earlier intervention and better treatment.

## Author contributions

PS, LM and CS conceptualized the topic and structure of the systematic review. LM and CS drafted and revised the manuscript. SN, AM, MA-A, CN-P, KN and PS provided expert opinion, edited, and approved the final manuscript. All authors contributed to the article and approved the submitted version.

## Funding

PS is funded by an NIHR Clinical Lectureship (CL-2018-17-002). This study was funded by the Fetal Medicine Foundation (KHN) (registered charity 1037116), National Institute for Health Research (NIHR) Biomedical Research Centre at Guy’s and St Thomas’ National Health Service Foundation Trust and King’s College London (IS-BRC-1215–20006). The views expressed in this Article are those of the authors and not necessarily those of the National Health Service, the NIHR, or the Department of Health.

## Conflict of interest

The authors declare that the research was conducted in the absence of any commercial or financial relationships that could be construed as a potential conflict of interest.

## Publisher’s note

All claims expressed in this article are solely those of the authors and do not necessarily represent those of their affiliated organizations, or those of the publisher, the editors and the reviewers. Any product that may be evaluated in this article, or claim that may be made by its manufacturer, is not guaranteed or endorsed by the publisher.
