# Supplementary material for: Chronic histiocytic intervillositis (CHI): current treatments and perinatal outcomes, a systematic review and a meta-analysis
Source: Front Endocrinol (Lausanne). 2022 Jul 22;13:945543. doi: 10.3389/fendo.2022.945543 (PMC9355722; doi:10.3389/fendo.2022.945543)
Supplement: Supplementary file 1 [file Table_1.docx]

**Histopathology: CHI and severity in the 4 studies included in the relevant meta-analysis**

**Parant**(Parant *et al.*, 2009)

A semi-quantitative grading was used according to the extent of chronic inflammatory infiltrate and the amount of fibrinoid deposition in the IVS. For each specimen, the slides with the most significant lesions were considered and evaluated as following: focal (when intervillous mononuclear infiltrate and fibrinoid deposition were localised in less than 10% of the slide), moderate (10–50% of the intervillous space of the slide was involved) or massive (more than 50% of the slide).

•*Moderate*: defined by focal or moderate intervillous lymphohistiocytic inflammatory infiltrate with mild or moderate fibrinoid deposition.

•*Severe*: defined by massive intervillous mononuclear inflammatory infiltrate with moderate or severe fibrinoid deposition. Massive and confluent perivillous fibrinoid deposition with mild mononuclear infiltrate was also considered in this level.

**Simula**(Simula *et al.*, 2020)

- Histology re-examined by a single pathologist
- Used Bos et al criteria to create grading system
- Low grade 5-49% intervillous space involvement
- High grade 50%+ intervillous space involvement
- Table shows in more detail (taken from paper)

| **Histopathological Features** |  |
| --- | --- |
| **Bos et al.[**[4](https://www.sciencedirect.com/science/article/pii/S0143400420302502?pes=vor#bib4)**] criteria for CIUE** | 1. Infiltrate present in 5% or more of the intervillous space 2. Approximately 80% of intervillous mononuclear cell infiltrate are histiocytes 3. No clinical or histological signs of an infectious process |
| **No CIUE (Grade 0)** | <5% of intervillous space involved by histiocytes and/or histiocyte-associated fibrin |
| **Low Grade (Grade 1)** | 5–49% of intervillous space involved by histiocytes and/or histiocyte-associated fibrin |
| **High Grade (Grade 2)** | 50% of intervillous space involved by histiocytes and/or histiocyte-associated fibrin |

**Sauvestre**(Sauvestre *et al.*, 2020)

- Reviewed by 3 pathologists
- Graded on % of macrophagic infiltrate in the IV space
- Grade 1=5-10%
- Grade 2= 10-50%
- Grade 3 = >50%

**Marchaudon**(Marchaudon *et al.*, 2011)

- Slides reread by 2 pathologists
- The actual grading is not specific- see below paragraph taken from paper

The intervillositis was considered diffuse when there were clusters of [histiocytes](https://www.sciencedirect.com/topics/medicine-and-dentistry/histiocyte) filling the entire intervillous space uniformly and massively at a magnification ×100 and was multifocal when the clusters were distributed heterogeneously in the intervillous spaces, varying from one microscopic field to another. The intensity of intervillositis was considered high when the clusters of histiocytes completely filled the intervillous space, moderate when the clusters were less voluminous, and low when they contained only a few histiocytes. The intensity of the fibrin deposits was considered high when they filled the intervillous spaces over several fields, moderate when these deposits were inconsistent, and low when there were only several scattered deposits.
